# Social Support and Prosocial Tendencies Among Chinese University Students: Parallel Indirect Associations Through Future Self-Continuity and Positive Empathy

**DOI:** 10.3390/bs16101760

**Published:** 2026-09-28

**Authors:** Biqin Li, Shunqi Yu, Bin Zhou, Gefei Zhang, Junhua Dang

**Affiliations:** 1 School of Psychology, Jiangxi Normal University, 99 Ziyang Avenue, Nanchang 330022, China; 2 Fuzhou Medical College, Nanchang University, No. 9 Donglin Road, Fuzhou 344000, China; 3 School of Humanities and Social Sciences, Xi’an Jiaotong University, No. 28 Xianning West Road, Xi’an 710049, China; 4 Department of Surgical Sciences, Uppsala University, Akademiska Sjukhuset, Entrance 70, 1tr, SE-751 85 Uppsala, Sweden

**Keywords:** social support, prosocial tendencies, future self-continuity, positive empathy, negative empathy, university students, parallel indirect associations

## Abstract

In the context of cultural change in China, an important developmental question is how attention to one’s own future relates to a willingness to benefit others. This cross-sectional study examined parallel indirect associations through future self-continuity and positive empathy in 4087 students from three universities in Jiangxi Province, China. Analyses adjusted for four demographic covariates and used percentile-bootstrap confidence intervals. Social support showed a positive total association with self-reported prosocial tendencies (*β* = 0.313). Specific indirect associations were positive through future self-continuity (*B* = 0.0057, 95% CI [0.0047, 0.0067]) and positive empathy (*B* = 0.0077, 95% CI [0.0066, 0.0089]), and a positive direct association remained. An exploratory comparison retaining future self-continuity found a larger indirect association through positive than negative empathy. These findings suggest that connections with the future self and responses to others’ positive experiences provide complementary perspectives on the association between social support and prosocial tendencies.

## 1. Introduction

Socioeconomic development and modernization have reshaped the value contexts in which young people develop, with greater emphasis on individual autonomy, self-expression, and personal goals in many societies (Santos et al., 2017). At the same time, in China, these orientations have not simply displaced longstanding emphases on social relationships and group-oriented values; a pluralist-constructivist perspective instead emphasizes their coexistence and integration (X. Chen, 2024). Within this value environment, youth development cannot be understood only in terms of whether individuals establish and pursue personal goals; it also concerns whether they remain socially connected and responsive to others while doing so. Prosocial tendencies capture one aspect of this other-oriented functioning, reflecting relatively stable inclinations to help, share, cooperate, and act for others’ benefit across situations (Wispé, 1972; Carlo & Randall, 2002). As personal goals become increasingly diverse and self-directed, understanding prosocial tendencies is relevant to how reciprocity, cooperation, and social connectedness are sustained alongside individual development. Evidence that prosocial attitudes, tendencies, and behaviors in China have changed over historical time further suggests that prosociality should not be treated as a fixed social background characteristic (S. Chen & Yang, 2025). Against this background, examining how the support university students receive relates to their willingness to benefit others offers a relational entry point to the broader question of how individual development and social connectedness are maintained together.

Supportive relationships provide emotional, informational, and instrumental resources through families, peers, teachers, and broader social networks. These resources can buffer stress and support psychological adaptation (Cohen & Wills, 1985; Brissette et al., 2002). A three-level meta-analysis found a positive association between social support and prosociality across 92 studies (Wang et al., 2024a). Longitudinal and experimental research has linked receiving support with subsequent prosocial motivation and behavior, with felt security examined as an explanatory process (Trombini et al., 2025). Among Chinese university students, Huang et al. (2023) also reported an indirect association between social support and prosocial behavior through positive affect. These two studies emphasize support recipients’ felt security and positive affect, respectively. Although these accounts identify important psychological correlates, they leave open how students’ connections with their own future and responses to others’ positive experiences jointly relate to prosocial tendencies within supportive relationships.

According to ecological systems theory, development unfolds within interacting social contexts. For university students, relationships with family members, peers, and teachers, together with the university setting, constitute important proximal contexts (Bronfenbrenner, 1979). Social support is provided, perceived, and mobilized within these interactions. Social-cognitive models of prosocial development complement this contextual perspective by examining cognitive and emotional characteristics alongside socialization experiences. For example, Carlo et al. (2018) reported indirect associations between parental or peer support and prosocial behavior involving perspective taking, empathic concern, and prosocial moral reasoning. Cross-cultural research has likewise examined the joint roles of cognitive and emotional characteristics in different forms of prosociality (Gülseven et al., 2020).

Future self-continuity refers to perceived connectedness between one’s present and future selves (Sokol & Serper, 2020). Its relevance here lies in asking whether connectedness to one’s own future is also associated with a willingness to benefit others. Support from significant others may give students encouragement and practical resources for pursuing future goals, providing a relational basis for connecting present efforts with anticipated roles. Consistent with this possibility, P. Guo et al. (2024) found a positive association between family support and future self-continuity among Chinese university students. Evidence concerning intertemporal choice, academic functioning, unethical behavior, and delinquency also shows that future self-continuity is relevant to present conduct (Bartels & Rips, 2010; Hershfield et al., 2012; Van Gelder et al., 2013; Adelman et al., 2017; Yang & Zeng, 2022). The relevance of this connection to present behavior can be understood from identity-based motivation: a future identity is more likely to guide action when it feels connected to the current self and relevant to that action (Nurra & Oyserman, 2018). Supportive relationships may therefore be associated with a stronger connection to the future self. When helping and cooperation are part of a valued future identity, this connection may also be relevant to present prosocial tendencies.

Empathy–altruism research has traditionally examined how empathic concern for another person’s distress motivates helping (Batson et al., 1981). Positive empathy broadens this focus to sharing others’ joy, success, or improvement (Andreychik & Migliaccio, 2015). Positive empathy specifically concerns sharing others’ positive experiences, whereas general positive affect does not, by itself, specify the object of those feelings. In dyadic research on sharing good news, listeners who shared more positive emotion also gave more supportive responses (Depow et al., 2026). Sharing positive events within supportive interactions may provide opportunities for students to attend to and share others’ positive emotions. In turn, sharing another person’s enjoyment or success may make efforts to create or sustain those outcomes more appealing. Morelli et al. (2015) reviewed evidence of positive associations between positive empathy, prosocial behavior, and social closeness. Positive empathy thus concerns the emotional significance of others’ positive experiences, complementing accounts centered on the support recipient’s own positive affect.

Negative empathy, or sharing others’ sadness and distress, provides a closely related comparison for understanding the positive-empathy association (Andreychik & Migliaccio, 2015; R. Guo et al., 2022). The two forms are related but have been associated with different motives: promoting others’ positive states versus reducing or preventing negative states (Andreychik & Lewis, 2017). Differences among empathy components have also been observed in social support–prosociality research, where perspective taking and empathic concern, but not personal distress, showed positive indirect associations in medical students (Wang et al., 2024b). An exploratory model including both forms of empathy can compare their associations with social support and prosocial tendencies, as well as the corresponding specific indirect associations.

These considerations place future self-continuity in a self-oriented temporal-cognitive domain and positive empathy in an other-oriented affective domain. The former concerns how students connect with their own future, whereas the latter concerns how they respond to other people’s positive experiences. Their joint examination asks whether a self-representation centered on one’s own future and a response to others’ positive experiences each carry information about prosocial tendencies within supportive relationships. The parallel model estimates each specific indirect association while accounting for the other construct and allowing their residuals to covary.

The present study therefore examined the total association between social support and self-reported prosocial tendencies and the specific indirect associations through future self-continuity and positive empathy in a parallel model. We hypothesized that (H1) social support would show a positive total association with self-reported prosocial tendencies; (H2) this association would include a positive specific indirect association through future self-continuity; and (H3) it would include a positive specific indirect association through positive empathy. To further examine the positive-empathy association, an exploratory model retained future self-continuity and compared the paths and specific indirect associations involving positive and negative empathy. A second exploratory analysis examined whether the two primary indirect associations were confined to particular forms of helping or were also evident across the six PTM subscales (Carlo & Randall, 2002).

Figure 1 shows the hypothesized model without estimated coefficients. The arrows denote predicted positive associations. The parallel model evaluates the two proposed indirect associations together while allowing future self-continuity and positive empathy to covary.

## 2. Materials and Methods

### 2.1. Participants and Procedure

This cross-sectional correlational questionnaire study used data collected in 2024 from students at three universities in Jiangxi Province, China. Recruitment was non-probability and convenience-based: surveys were organized through classes, and university counselors distributed a link hosted on Wenjuanxing (Changsha Ranxing Information Technology Co., Ltd., Changsha, China). Participation was voluntary. Students who chose to participate received a small amount of extra credit toward their regular course grade. A total of 4500 questionnaire submissions were initially recorded. The focal measures were embedded in a broader project questionnaire.

Responses were excluded if completion of the full questionnaire took less than 480 s or more than 5000 s, if either of the two instructed-response attention checks was answered incorrectly, or if screening identified highly patterned or internally inconsistent responding across selected positively and reverse-worded items. After screening and preparation of the analytic dataset, 4087 complete records were retained. Participants were aged 17–25 years (*M* = 20.31, *SD* = 1.28); 1300 were male and 2787 female; 3090 reported rural and 997 urban residence; and 428 were only children and 3659 non-only children.

The study was reviewed and approved by the Ethics Committee of the School of Psychology, Jiangxi Normal University (protocol code IRB-JXNU-PSY-2024033), and data collection was completed within the approved project period. Before accessing the questionnaire, participants viewed an electronic informed-consent statement describing the research purpose, voluntary participation, academic use of the data, and the right to discontinue before submission; they proceeded only after indicating consent. The analytic dataset was anonymized before analysis and contained no directly identifying information.

### 2.2. Measures

#### 2.2.1. Social Support

Social support was assessed with a university-context adaptation of the 10-item Social Support Rating Scale (SSRS; Xiao, 1994), which covers subjective support, objective support, and support utilization. A previous Chinese validation study reported that these three factors explained 55.84% of the variance, with subscale–total correlations of 0.724–0.835 and subscale *α* values of 0.825–0.849 (Liu et al., 2008). In the administered version, neighbors and coworkers were replaced by roommates and classmates, and support-source options were adapted to student relationships and organizations. A representative item asks, ‘How many close friends do you have from whom you can obtain support and assistance?’ Items use specific response formats rather than one common Likert scale. Item 5 was the sum of the family-support entries. Items 6 and 7 counted the selected support sources, with ‘no source’ contributing zero. The additional ‘other’ option in item 3 was coded as 4 (41 records). Sensitivity analyses examined alternative scoring of these responses and exclusion of the corresponding cases; details are provided in the accompanying sensitivity-analysis documentation. The 10 item-specific scores were summed. Higher totals indicate more received and perceived support and greater support utilization. In this sample, *M* = 38.079, *SD* = 6.057, and Cronbach’s *α* = 0.712.

#### 2.2.2. Prosocial Tendencies

Self-reported prosocial tendencies were measured with the Chinese 26-item Prosocial Tendencies Measure (PTM; Carlo & Randall, 2002; Kou et al., 2007). Its six dimensions are public, anonymous, altruistic, compliant, emotional, and dire tendencies. In the Chinese validation, the six-factor model fit the data well (*χ*^2^/df = 1.97, CFI = 0.99, TLI = 0.98, RMSEA = 0.06), subscale–total correlations ranged from 0.49 to 0.80, and subscale *α* values ranged from 0.56 to 0.78 (Kou et al., 2007). A representative item is ‘When others ask me for help, I seldom refuse.’ Items are rated from 1 (does not describe me at all) to 5 (describes me very well). Because the primary question concerns a broad prosocial tendency rather than differences among forms of helping, the 26-item mean was retained as the primary composite. The six established subscale means were examined separately in exploratory analyses. In this sample, overall *α* = 0.945 and subscale *α* values ranged from 0.743 to 0.843. Higher scores indicate stronger self-reported prosocial tendencies.

#### 2.2.3. Future Self-Continuity

Future self-continuity was assessed with the 10-item Future Self-Continuity Questionnaire (Sokol & Serper, 2020; Shen et al., 2022). It measures similarity, vividness, and positivity; for example, respondents evaluate the perceived similarity between their current self and their self 10 years in the future. Items are rated from 1 (completely uncharacteristic of me) to 6 (completely characteristic of me). The original authors calculated the overall score as the mean of the 10 items. Validation among Chinese university students supported the three-factor structure (CFI = 0.964, TLI = 0.950, RMSEA = 0.060). The total score correlated with the Future Self-Continuity Scale and the Consideration of Future Consequences Scale (*r* = 0.31 and 0.73, respectively), and two-week test–retest reliability was 0.80 (Shen et al., 2022). In this sample, *α* = 0.879. Higher means indicate stronger future self-continuity.

#### 2.2.4. Positive and Negative Empathy

All 14 items of the Chinese Positive and Negative Empathy Scale were administered (PaNES; Andreychik & Migliaccio, 2015; R. Guo et al., 2022), with seven items assessing each form of empathy. Items are rated from 1 (completely uncharacteristic) to 5 (completely characteristic) and averaged within each subscale. Representative items are ‘When other people receive good news, I am happy for them’ and ‘When a friend is sad, I become sad too.’ Chinese validation supported the two-factor structure; *α* values were 0.866 and 0.798 and two-week test–retest correlations were 0.697 and 0.604 for positive and negative empathy, respectively. Correlations with the six PTM subscales ranged from 0.299 to 0.502 for positive empathy and from 0.301 to 0.474 for negative empathy (R. Guo et al., 2022). In this sample, *α* = 0.851 and 0.859, respectively. Positive empathy was included in the primary model, and negative empathy was added in the exploratory comparison.

### 2.3. Data Analysis

Analyses were performed in R 4.6.1 using 4087 complete cases. The adapted SSRS total was calculated with item-specific scoring; the FSCQ, PaNES subscales, overall PTM, and PTM subscales were item means. The primary analyses included descriptive statistics, Cronbach’s *α*, Pearson correlations, a 53-item Harman single-factor diagnostic, Welch *t*-tests, age-group ANOVAs, and variance inflation factors. Age, gender, residence, and only-child status were retained as a basic demographic adjustment set rather than selected according to preliminary significance tests. H1 was tested with a regression of prosocial tendencies on the SSRS total and these covariates. Hierarchical regression entered the four demographics at Step 1, social support at Step 2, and future self-continuity plus positive empathy at Step 3 to estimate *R*^2^ and Δ*R*^2^.

The primary parallel indirect-association model in lavaan 0.6–21 included social support as the predictor, self-reported prosocial tendencies as the outcome, and future self-continuity and positive empathy simultaneously. Their residual covariance was freely estimated. The same four demographic covariates were included in each regression equation. Sensitivity analyses without demographic covariates and without only-child status assessed whether the focal conclusions changed under alternative specifications. The primary analysis requested 5000 bootstrap resamples and reported 95% percentile-bootstrap confidence intervals. Parameter tables present *z*-based *p*-values using bootstrap standard errors. Because the model was saturated, global fit indices were not interpreted. The paths describe cross-sectional statistical associations rather than causal mediation (Hayes, 2018).

Two exploratory analyses used the same sample, covariates, and bootstrap procedure. First, negative empathy was added alongside future self-continuity and positive empathy, with residual covariances freely estimated among all three. Three unstandardized contrasts compared the social-support paths, the conditional paths to PTM total, and the specific indirect associations through positive and negative empathy. Second, six models replaced PTM total with one of its six subscales, retaining future self-continuity and positive empathy in parallel. Benjamini–Hochberg correction was applied separately to the three empathy contrasts and the 12 subscale-specific indirect associations (Benjamini & Hochberg, 1995). The empathy-comparison model had 4991 valid bootstrap resamples; the six subscale models had 4986–5000. Standardized coefficients summarize effect magnitudes; the contrast confidence intervals and adjusted *p*-values refer to the unstandardized differences.

## 3. Results

### 3.1. Descriptive Statistics

Table 1 reports categorical characteristics as n and % and continuous variables as *M*, *SD*, skewness, and kurtosis. Mean scores were 38.08 (*SD* = 6.06) for social support, 4.12 (*SD* = 0.69) for future self-continuity, 3.74 (*SD* = 0.52) for positive empathy, and 3.77 (*SD* = 0.47) for prosocial tendencies. Negative empathy, used in the exploratory model, had a mean of 3.29 (*SD* = 0.63).

### 3.2. Common-Method Diagnostic

As a preliminary common-method diagnostic, an unrotated principal-components analysis of the 53 focal items yielded 11 components with eigenvalues greater than 1; the first accounted for 26.02% of the variance.

### 3.3. Correlation Analysis

Table 2 presents correlations among the primary-model variables and demographic covariates. The SSRS total was positively correlated with positive empathy (*r* = 0.315), future self-continuity (*r* = 0.317), and prosocial tendencies (*r* = 0.311), all *ps* < 0.001. Positive empathy and future self-continuity were also correlated with prosocial tendencies (*r* = 0.424 and 0.361, respectively, *ps* < 0.001).

### 3.4. Demographic Differences

Female students reported higher social support, positive empathy, and future self-continuity than male students; the corresponding Welch’s *t* values were −4.668, −8.221, and −4.078, respectively, all *ps* < 0.001. Urban students reported slightly higher future self-continuity than rural students (*t* = −2.221, *p* = 0.026). Age-group ANOVAs were significant for prosocial tendencies, *F*(8, 4078) = 2.037, *p* = 0.039, and positive empathy, *F*(8, 4078) = 1.975, *p* = 0.046. These group differences were small and are reported descriptively.

### 3.5. Total and Parallel Indirect Associations

H1 was supported: adjusting for the four demographics, social support showed a positive total association with prosocial tendencies, *B* = 0.024, *SE* = 0.001, *β* = 0.313, 95% CI [0.022, 0.026], *p* < 0.001. All VIFs were ≤1.192. In the parallel model, social support was associated with future self-continuity (*B* = 0.036, *β* = 0.314) and positive empathy (*B* = 0.027, *β* = 0.308); future self-continuity (*B* = 0.160, *β* = 0.235) and positive empathy (*B* = 0.289, *β* = 0.325) were associated with prosocial tendencies; and the remaining direct association was *B* = 0.011, *β* = 0.139 (all *ps* < 0.001; Table 3). The mediator residual covariance was 0.055, 95% CI [0.043, 0.067], *p* < 0.001 (residual r = 0.171). Demographics alone explained 0.4% of outcome variance; adding social support increased *R*^2^ by 0.097, and adding both mediators increased *R*^2^ by 0.166. The full outcome *R*^2^ was 0.268; mediator *R*^2^ values were 0.105 and 0.116. Figure 2 presents the fully standardized focal paths.

The confidence intervals for both specific indirect associations excluded zero: future self-continuity, *B* = 0.0057, 95% CI [0.0047, 0.0067], and positive empathy, *B* = 0.0077, 95% CI [0.0066, 0.0089] (Table 4). The total indirect association was 0.0134, 95% CI [0.0119, 0.0149]. Conclusions were unchanged without covariates (specific indirect Bs = 0.0057 and 0.0078; direct *B* = 0.0104) and without only-child status (0.0057, 0.0077, and 0.0107, respectively); all corresponding 95% intervals excluded zero.

### 3.6. Exploratory Analyses

#### 3.6.1. Comparison of Positive and Negative Empathy

In the exploratory model retaining future self-continuity, social support was associated with positive empathy (*B* = 0.0266, *β* = 0.308, *p* < 0.001) and negative empathy (*B* = 0.0044, *β* = 0.042, *p* = 0.013). The unstandardized path difference favored positive empathy (*B* = 0.0222, 95% CI [0.0192, 0.0253], BH-adjusted *p* < 0.001). Both empathy scores were positively associated with PTM total after adjustment, with a larger coefficient for positive empathy (difference *B* = 0.1445, 95% CI [0.0872, 0.2032], BH-adjusted *p* < 0.001). The specific indirect associations were *B* = 0.0061, 95% CI [0.0050, 0.0074], through positive empathy and *B* = 0.0004, 95% CI [0.0001, 0.0007], through negative empathy (unadjusted *p* = 0.024 for the latter). Their difference was *B* = 0.0057, 95% CI [0.0045, 0.0070], BH-adjusted *p* < 0.001 (Table 5). The indirect association through future self-continuity remained positive (*B* = 0.0059, 95% CI [0.0049, 0.0070]). Adding negative empathy increased outcome *R*^2^ from 0.268 to 0.277.

#### 3.6.2. Associations Across PTM Subscales

All 12 specific indirect associations through future self-continuity and positive empathy were positive across the public, anonymous, altruistic, compliant, emotional, and dire subscales. Their 95% bootstrap confidence intervals excluded zero, and all BH-adjusted *p*-values were below 0.001 (Table 6). Fully standardized indirect estimates ranged from 0.053 to 0.070 through future self-continuity and from 0.069 to 0.098 through positive empathy. Outcome *R*^2^ values ranged from 0.143 for anonymous tendencies to 0.241 for emotional tendencies. Thus, same-direction associations were observed across all six forms of prosocial tendency in this sample.

## 4. Discussion

Social support was positively associated with self-reported prosocial tendencies. The specific indirect associations through future self-continuity and positive empathy remained positive when estimated together, and a positive direct association remained. The exploratory comparison showed a larger indirect association through positive than negative empathy, although both were positive. The two primary indirect associations were also observed across all six PTM subscales.

Social-cognitive models of prosocial development emphasize both cognitive and emotional characteristics in the relation between socialization experiences and helping (Carlo et al., 2018; Gülseven et al., 2020). The present findings are consistent with this approach and extend its application to a self-oriented temporal-cognitive domain and an other-oriented affective domain. Students’ connections with a future identity and their emotional responses to others’ positive experiences thus provide complementary perspectives on the association between social support and prosocial tendencies. This individual-level perspective is relevant to the broader concern with personal goals and social connectedness in Chinese developmental research (X. Chen, 2024).

Future self-continuity was positively associated with prosocial tendencies even when positive empathy was included in the model. Family support has been associated with stronger future self-continuity in university students (P. Guo et al., 2024), and support may provide encouragement and resources for maintaining future goals. From an identity-based motivation perspective, continuity with a valued future identity can make identity-relevant choices meaningful in the present (Nurra & Oyserman, 2018). For students who envisage helping and cooperation as part of their future roles, this may be one reason that future self-continuity accompanies stronger prosocial tendencies. Directly measuring the content of students’ future identities would help test this interpretation. Longitudinal research in nursing students has also reported phase-dependent and asymmetric prospective associations between social support and future self-continuity (Lv et al., 2026), highlighting the need to examine their development over time.

Positive empathy was also positively associated with prosocial tendencies after future self-continuity was included in the model. Earlier research examined positive affect within the social support–prosociality association (Huang et al., 2023); the present study instead measured positive emotion shared in response to other people’s experiences. The exploratory comparison further showed that both empathy scores retained positive conditional associations with prosocial tendencies, but the social-support path and specific indirect association were larger for positive empathy. This pattern complements work distinguishing other empathy components in support–prosociality models (Wang et al., 2024b) and is consistent with the different motives associated with positive and negative empathy (Andreychik & Lewis, 2017). It directs attention to students’ responses to others’ enjoyment, progress, and success alongside their responses to suffering.

Both primary indirect associations were positive across the six PTM subscales. The pattern was evident in helping tendencies that differ in visibility, responsiveness to requests, emotional salience, and urgency (Carlo & Randall, 2002). These results extend the description beyond the overall PTM score and show that same-direction associations were observable across several forms of helping within this sample.

At a practical level, universities could consider peer mentoring and student-support networks to make support more accessible. Service-learning research has linked structured reflection and community involvement with better student outcomes, including civic engagement (Celio et al., 2011). Within such activities, reflection could connect present helping with responsibilities in students’ anticipated family, community, or professional roles. Students could also consider the concrete positive changes experienced by beneficiaries, so that reflection attends to others’ outcomes as well as to the students’ own learning. Future intervention studies could evaluate whether these activities strengthen future self-continuity, positive empathy, and prosocial engagement.

## 5. Limitations and Future Directions

The cross-sectional design limits conclusions about temporal ordering and model interpretation. Because all focal variables were measured concurrently, their temporal ordering cannot be determined. Positioning social support as the predictor and prosocial tendencies as the outcome reflects the theoretical framing and research question of the present study rather than empirically established temporal precedence. Reverse or reciprocal relationships remain plausible, including the possibility that prosocial tendencies may influence perceived social support or empathic responses. Accordingly, the observed indirect associations should not be interpreted as evidence that future self-continuity or positive empathy constitute causal intervening processes, nor do they establish that the proposed structure is preferable to other plausible psychological explanations. Longitudinal studies with repeated assessments of the focal constructs, together with experimental research and direct comparisons of alternative pathways, are needed to clarify these relationships.

The characteristics of the present sample also limit the generalizability of the findings. Participants were recruited by convenience sampling from three universities in Jiangxi Province, and women were overrepresented. Cultural value orientations were not measured, and the design does not test the effects of cultural change. Reciprocity norms, relationship expectations, and support-seeking practices may vary across settings, making replication in other regions and cultures important.

The reliance on self-report measures introduces additional limitations. The PTM assesses prosocial tendencies rather than directly observed helping behavior. The single-factor diagnostic does not rule out shared-method bias. Future research could combine self-report data with behavioral tasks or reports from other informants.

The use of composite scores from multidimensional instruments also limits the specificity of the findings. The empathy comparison and PTM subscale analyses were exploratory, and the correlated subscales were assessed in the same participants. Independent samples and latent measurement models could test the robustness of these patterns and more specific hypotheses about similarities and differences among dimensions.

Situational conditions that may shape these associations were not included in the present model. Future studies could examine time pressure, helping costs, salient social norms, face concerns, and the presence of others as possible moderators, using both behavioral and self-report outcomes.

## 6. Conclusions

In this sample of Chinese university students, social support was positively associated with self-reported prosocial tendencies, with specific indirect associations through future self-continuity and positive empathy. The two constructs capture a self-oriented temporal-cognitive domain and an other-oriented affective domain that can be considered together in research on supportive relationships. In the exploratory model, the indirect association through positive empathy was larger than that through negative empathy; the primary associations were also positive across six forms of prosocial tendency. These findings highlight both connections with the future self and emotional responses to others’ positive experiences in understanding the support–prosociality association.

## Figures and Tables

**Figure 1:**
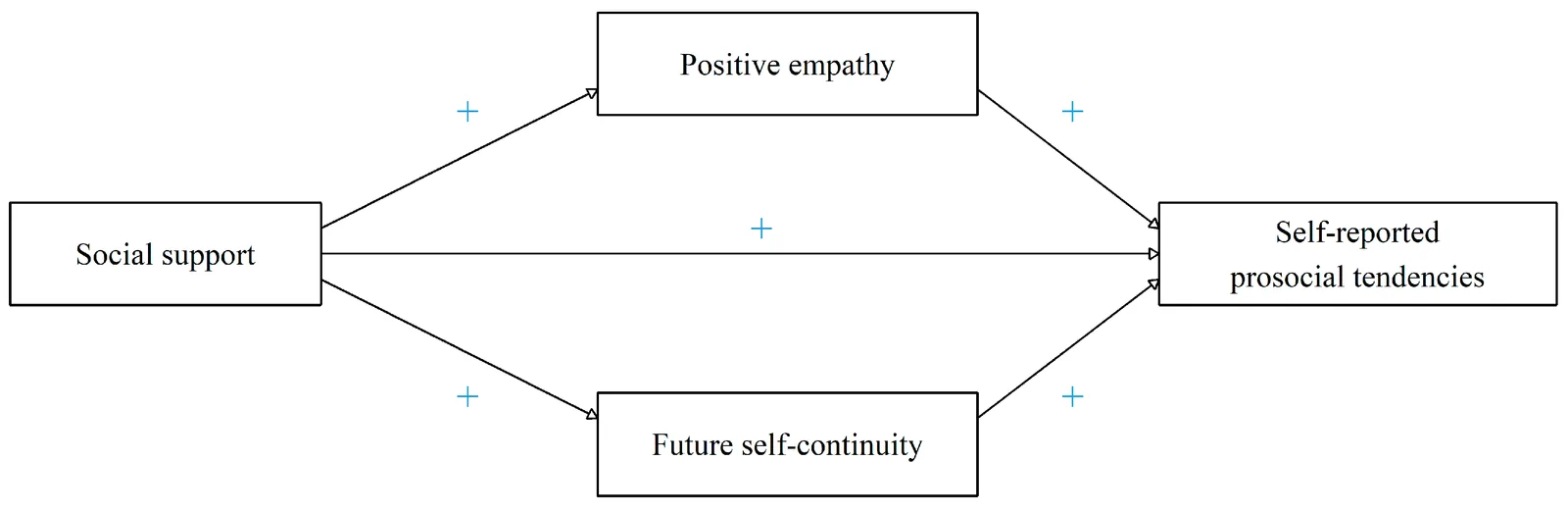
Hypothesized parallel indirect-association model linking social support with self-reported prosocial tendencies through future self-continuity and positive empathy. All focal paths were predicted to be positive. Demographic covariates are omitted for clarity.

**Figure 2:**
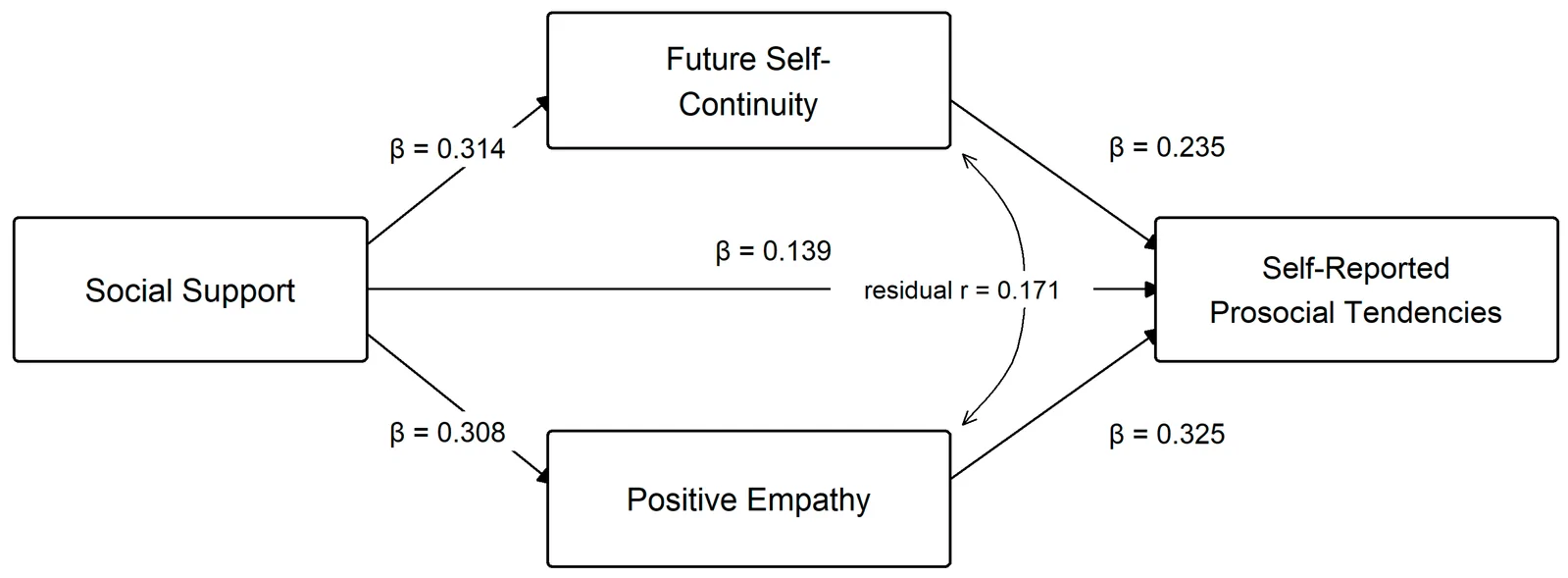
Adjusted parallel indirect-association model with fully standardized coefficients for the focal paths. Age, gender, residence, and only-child status were included as covariates but are omitted for clarity. The curved double-headed arrow represents the residual correlation between future self-continuity and positive empathy. All displayed focal paths were significant at *p* < 0.001.

**Table 1:** Sample characteristics and descriptive statistics.

Panel A. Categorical variables				
**Variable**	**Category**		**n**	**%**
Gender	Male		1300	31.81
	Female		2787	68.19
Residence	Rural		3090	75.61
	Urban		997	24.39
Only-child status	Only child		428	10.47
	Non-only child		3659	89.53
Panel B. Continuous variables				
**Variable**	***M***	***SD***	**Skewness**	**Kurtosis**
Age	20.31	1.28	0.40	−0.13
Social support (SSRS total)	38.08	6.06	−0.19	−0.17
Future self-continuity	4.12	0.69	−0.45	1.18
Positive empathy	3.74	0.52	−0.61	1.99
Negative empathy	3.29	0.63	−0.38	0.51
Prosocial tendencies	3.77	0.47	0.21	1.28

Note. Categorical variables are reported as n and %. Continuous variables are reported as *M*, *SD*, skewness, and kurtosis. Negative empathy is used in the exploratory model. SSRS = Social Support Rating Scale.

**Table 2:** Correlations among study variables.

	1	2	3	4	5	6	7	8
1. Gender	—							
2. Age	−0.048 **	—						
3. Residence	−0.006	−0.092 ***	—					
4. Only-child status	0.094 ***	−0.006	−0.288 ***	—				
5. Positive empathy	0.137 ***	0.051 **	−0.015	0.017	—			
6. Future self-continuity	0.067 ***	0.016	0.035 *	−0.016	0.258 ***	—		
7. Social support (SSRS total)	0.075 ***	−0.015	−0.012	0.030	0.315 ***	0.317 ***	—	
8. Prosocial tendencies	0.018	0.051 **	0.027	0.002	0.424 ***	0.361 ***	0.311 ***	—

Note. Gender was coded as 1 = male and 2 = female; residence as 1 = rural and 2 = urban; and only-child status as 1 = only child and 2 = non-only child. * *p* < 0.05, ** *p* < 0.01, *** *p* < 0.001.

**Table 3:** Path coefficients in the parallel indirect-association model.

Outcome	Predictor	*B*	*SE*	95% CI	*β*	*z*	*p*
Future self-continuity	Social support (SSRS total)	0.036	0.002	[0.032, 0.039]	0.314	19.444	<0.001
Future self-continuity	Age	0.014	0.008	[−0.002, 0.030]	0.026	1.723	0.085
Future self-continuity	Gender	0.069	0.024	[0.023, 0.116]	0.047	2.938	0.003
Future self-continuity	Residence	0.058	0.025	[0.009, 0.108]	0.036	2.307	0.021
Future self-continuity	Only-child status	−0.043	0.035	[−0.112, 0.024]	−0.019	−1.216	0.224
Positive empathy	Social support (SSRS total)	0.027	0.001	[0.024, 0.029]	0.308	19.250	<0.001
Positive empathy	Age	0.025	0.006	[0.013, 0.037]	0.061	4.013	<0.001
Positive empathy	Gender	0.132	0.018	[0.097, 0.167]	0.117	7.375	<0.001
Positive empathy	Residence	−0.007	0.019	[−0.044, 0.030]	−0.006	−0.370	0.711
Positive empathy	Only-child status	−0.008	0.028	[−0.063, 0.048]	−0.005	−0.290	0.772
Prosocial tendencies	Social support (SSRS total)	0.011	0.001	[0.008, 0.013]	0.139	8.831	<0.001
Prosocial tendencies	Future self-continuity	0.160	0.012	[0.136, 0.183]	0.235	13.208	<0.001
Prosocial tendencies	Positive empathy	0.289	0.017	[0.257, 0.322]	0.325	17.489	<0.001
Prosocial tendencies	Age	0.012	0.005	[0.002, 0.022]	0.033	2.375	0.018
Prosocial tendencies	Gender	−0.052	0.014	[−0.079, −0.025]	−0.052	−3.765	<0.001
Prosocial tendencies	Residence	0.034	0.016	[0.003, 0.065]	0.031	2.119	0.034
Prosocial tendencies	Only-child status	0.016	0.023	[−0.030, 0.059]	0.010	0.690	0.490

Note. *B* = unstandardized coefficient; *β* = fully standardized coefficient. Percentile-bootstrap confidence intervals are shown. Age, gender, residence, and only-child status were included as adjustments. *R*^2^ = 0.105 for future self-continuity, 0.116 for positive empathy, and 0.268 for prosocial tendencies.

**Table 4:** Total, direct, and indirect associations in the parallel model.

Association	*B*	*SE*	*95%* CI	*β*
Indirect via future self-continuity	0.0057	0.0005	[0.0047, 0.0067]	0.074
Indirect via positive empathy	0.0077	0.0006	[0.0066, 0.0089]	0.100
Total indirect association	0.0134	0.0008	[0.0119, 0.0149]	0.174
Direct association	0.0107	0.0012	[0.0083, 0.0131]	0.139
Total association	0.0241	0.0012	[0.0217, 0.0265]	0.313

Note. *B* = unstandardized coefficient; *β* = fully standardized coefficient. Confidence intervals are 95% percentile-bootstrap intervals from 5000 requested resamples. All *p* values were below 0.001.

**Table 5:** Comparison of positive and negative empathy in the exploratory model.

Association	*B*/Difference	*SE*	95% CI	β	*p*	*p*_BH_
Social support → positive empathy	0.0266	0.0014	[0.0238, 0.0294]	0.308	<0.001	—
Social support → negative empathy	0.0044	0.0018	[0.0010, 0.0079]	0.042	0.013	—
Positive empathy → PTM total	0.2291	0.0195	[0.1905, 0.2675]	0.258	<0.001	—
Negative empathy → PTM total	0.0845	0.0141	[0.0565, 0.1123]	0.115	<0.001	—
Indirect via positive empathy	0.0061	0.0006	[0.0050, 0.0074]	0.079	<0.001	—
Indirect via negative empathy	0.0004	0.0002	[0.0001, 0.0007]	0.005	0.024	—
PE − NE: social-support path	0.0222	0.0015	[0.0192, 0.0253]	—	—	<0.001
PE − NE: PTM conditional path	0.1445	0.0297	[0.0872, 0.2032]	—	—	<0.001
PE − NE: indirect association	0.0057	0.0006	[0.0045, 0.0070]	—	—	<0.001

Note. PE = positive empathy; NE = negative empathy; PTM = Prosocial Tendencies Measure. Future self-continuity and the four demographic covariates remained in the model; residual covariances among the three intermediate variables were freely estimated. *p* is unadjusted; *p*_BH_ is Benjamini–Hochberg adjusted across the three PE–NE contrasts. Confidence intervals and *p* values test *B* or the unstandardized difference, not the displayed standardized coefficients. For contrast rows, β is omitted. There were 4991 valid bootstrap resamples.

**Table 6:** Indirect associations across the six PTM subscales.

PTM Subscale	α	*M* (*SD*)	Indirect via FSC: *B* [95% CI]; β	Indirect via PE: *B* [95% CI]; β	*R* ^2^
Public	0.785	3.55 (0.63)	0.0058 [0.0045, 0.0070] β = 0.056	0.0088 [0.0074, 0.0103] β = 0.085	0.172
Anonymous	0.843	3.69 (0.60)	0.0057 [0.0045, 0.0070] β = 0.058	0.0068 [0.0056, 0.0082] β = 0.069	0.143
Altruistic	0.825	3.99 (0.52)	0.0061 [0.0050, 0.0072] β = 0.070	0.0064 [0.0052, 0.0076] β = 0.073	0.215
Compliant	0.826	3.69 (0.56)	0.0049 [0.0038, 0.0061] β = 0.053	0.0082 [0.0070, 0.0096] β = 0.090	0.179
Emotional	0.835	3.78 (0.56)	0.0063 [0.0051, 0.0075] β = 0.068	0.0090 [0.0077, 0.0104] β = 0.098	0.241
Dire	0.743	4.00 (0.49)	0.0054 [0.0044, 0.0065] β = 0.067	0.0063 [0.0052, 0.0075] β = 0.078	0.182

Note. FSC = future self-continuity; PE = positive empathy; PTM = Prosocial Tendencies Measure. All models included age, gender, residence, and only-child status. BH correction was applied to the family of 12 specific indirect associations; all adjusted *p*-values were below 0.001. Confidence intervals are 95% percentile-bootstrap intervals. Valid resamples, in table order, were 5000, 4998, 4996, 4998, 4997, and 4986.

## Data Availability

The anonymized analytic dataset, variable codebook, R/lavaan analysis code, statistical outputs, and data-screening documentation supporting this study are openly available in the Open Science Framework (OSF) at https://osf.io/ujafw/ accessed on 9 September 2026. The shared analytic files contain no directly identifying information.
